# High-performance computing service for bioinformatics and data science

**DOI:** 10.5195/jmla.2018.512

**Published:** 2018-10-01

**Authors:** Jean-Paul Courneya, Alexa Mayo

**Affiliations:** Bioinformationist, Health Sciences and Human Services Library, University of Maryland, Baltimore, MD 21201; Associate Director for Services, Health Sciences and Human Services Library, University of Maryland, Baltimore, MD 21201

## Abstract

Despite having an ideal setup in their labs for wet work, researchers often lack the computational infrastructure to analyze the magnitude of data that result from “-omics” experiments. In this innovative project, the library supports analysis of high-throughput data from global molecular profiling experiments by offering a high-performance computer with open source software along with expert bioinformationist support. The audience for this new service is faculty, staff, and students for whom using the university’s large scale, CORE computational resources is not warranted because these resources exceed the needs of smaller projects. In the library’s approach, users are empowered to analyze high-throughput data that they otherwise would not be able to on their own computers. To develop the project, the library’s bioinformationist identified the ideal computing hardware and a group of open source bioinformatics software to provide analysis options for experimental data such as scientific images, sequence reads, and flow cytometry files. To close the loop between learning and practice, the bioinformationist developed self-guided learning materials and workshops or consultations on topics such as the National Center for Biotechnology Information’s BLAST, Bioinformatics on the Cloud, and ImageJ. Researchers apply the data analysis techniques that they learned in the classroom in the library’s ideal computing environment.

Despite having an ideal setup in their labs for wet work, researchers often lack the computational infrastructure to analyze the magnitude of data that results from genomics, transcriptomics, and proteomics experiments. To address this need, the Health Sciences and Human Services Library at the University of Maryland, Baltimore, has developed an innovative program supporting analysis of high-throughput data from global molecular profiling experiments by offering a high-performance computer (HPC) with open source software, along with expert bioinformationist support. The intended audience for this new service is faculty, staff, and students for whom using the university’s large scale, CORE computational resources is not warranted. In the library’s approach, users are empowered to analyze high-throughput data beyond what they would be able to do on their own computers.

The library began this project by responding to an internal, university request for funding projects to support faculty, staff, and students and received notice of funding in the summer of 2017. The bioinformationist then consulted with campus and external experts and colleagues to research hardware and software options for the HPC. Software and hardware were acquired, installed, and configured with assistance from the library’s information technology network administrator. In the fall of 2017, rigorous testing was performed to ensure the hardware and software were operational to expectations. As a result of this testing, a network-attached storage device was added to back up the drives and data that run the HPC. The new service was launched in late fall 2017 and promoted throughout the university.

The HPC has state-of-the-art hardware and commercial and open source software for performing analysis of experimental data, such as scientific images, sequence reads, and flow cytometry files (Figure 1). The HPC includes a Dell Tower with an 8-core Intel Xeon E5-2667 v4 @3.20GHz CPU, which is the same type of processor used in most data centers. The HPC has 128GB of RAM and an 8TB solid state hard drive for running the virtual machines.

**Figure 1:**
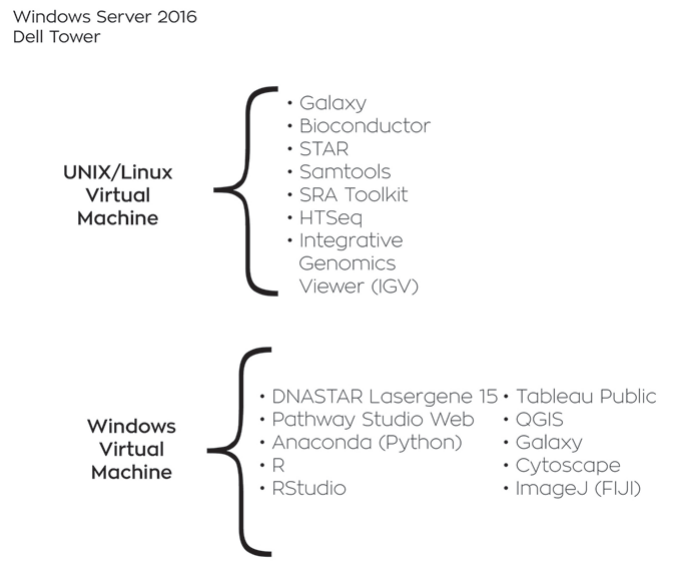
System profile

This project demonstrates the importance of using bioinformatics tools for closing the loop between learning and practice. This was accomplished by the bioinformationist developing self-guided learning materials and workshops or consultations on topics such as downloading publicly available sequence data, the National Center for Biotechnology Information’s BLAST, command line for Genomics, and ImageJ. Researchers then can apply the data analysis techniques that they learned in the classroom in an ideal computing environment with expert support.

The HPC has opened up opportunities for new collaborations on campus. The CORE imaging facility recently included the library as a collaborator for data analysis in a proposal to acquire a 3D electron microscope (EM). Because the processing and analysis of 3D EM data requires massive computing resources, the proposal for the microscope included the contributions of library resources and expertise in justifying the funding of the instrument.

One limitation currently experienced is the ability to provide remote access to the HPC. Because there are huge demands on researchers’ time, they tend to try to optimize how and where they spend it. Sometimes leaving the lab poses a significant drop in productivity. The bioinformationist is considering how to open up some or all of the resources of the HPC via remote user access. This raises some security and software licensing issues, which would complicate the ease of providing access. The library will continue to push the boundaries of the HPC service to ensure the service remains innovative and useful to the faculty, staff, and students at the university.

